# Heterogeneous duplication and point mutation of *ace-1* confer organophosphates and carbamates resistance to *Anopheles sinensis* field populations in Guizhou Province, China

**DOI:** 10.1186/s40249-026-01421-0

**Published:** 2026-03-09

**Authors:** Qiu-Qiu Xiao, Wei-Yi Li, Qiu-Guo Liang, Hai-Mo Shen, Jin-Zhi Cheng, Xi Yang, Jun-Hu Chen, Jia-Hong Wu

**Affiliations:** 1 Guizhou Key Laboratory of Microbiology and Infectious Disease Prevention and Control, Department of Medical Parasitology, School of Basic Medical Sciences, Guizhou Medical University, Guiyang, 550025 China; 2 Department of Basic Medical Sciences, Qiannan Medical College for Nationalities, Duyun, 558000 China; 3 National Key Laboratory of Intelligent Tracking and Forecasting for Infectious Diseases, National Institute of Parasitic Diseases, Chinese Center for Diseases Control and Prevention (Chinese Center for Tropical Diseases Research), Shanghai, 200025 China; 4 NHC Key Laboratory of Parasite and Vector Biology, WHO Collaborating Center for Tropical Diseases, Shanghai, 200025 China; 5 National Center for International Research on Tropical Diseases, Shanghai, 200025 China; 6 Hainan Tropical Diseases Research Center (Hainan Sub-Center, Chinese Center for Tropical Diseases Research), Haikou, 571199 China

**Keywords:** *Anopheles sinensis*, *Ace-1*, Heterogeneous duplication, Mutation, Insecticide resistance

## Abstract

**Background:**

Guizhou Province has historically been a region severely affected by malaria in China. For decades, vector control has served as a cornerstone of national efforts to control and eliminate malaria. However, the efficacy of this strategy is largely challenged by the development of insecticide resistance. In the present study, the resistance status to organophosphates (OPs) and carbamates (CBs) of *Anopheles sinensis* field populations across Guizhou Province was investigated with a primary focus on elucidating the underlying mechanisms.

**Methods:**

From 2017 to 2024, mosquitoes were collected intermittently using mosquito-killing lamps across Guizhou Province. *An. sinensis* specimens were identified using morphological and molecular methods. Subsequently, we genotyped the *ace-1* gene via PCR and measured acetylcholinesterase1 (AChE1) residual activity using biochemical assays. Whole-genome sequencing of individual mosquitoes was performed using Illumina sequencing, and the copy number of the *ace-1* gene was quantified by standard genomic DNA quantitative PCR. Two independent-sample t-test and a chi-squared test had been used in this study.

**Results:**

Female *An. sinensis* were collected from 12 field populations across Guizhou Province. Following species identification, point mutations in the *ace-1* gene were detected in 551 mosquito samples. Only one point mutation, G119S, was identified across all populations, with the frequency of the *ace-1* mutant genotypes (119GS and 119SS) exceeding 66% in 11 out of the 12 populations. Heterozygotes were the predominant genotype. The AChE1 activity was not inhibited by propoxur in 10 populations. A significant departure from Hardy–Weinberg equilibrium was observed in 6 of the 12 populations, indicating an excess of heterozygotes in these populations. Notably, heterogeneous duplication of the *ace-1* gene in *An. sinensis* was detected for the first time through genomic scanning and *ace-1* copy number quantification.

**Conclusions:**

Resistance to OPs and CBs is widespread in *An. sinensis* populations across Guizhou Province. Both heterogeneous duplication and point mutation of the *ace-1* gene in *An. sinensis* likely contribute to resistance to OPs and CBs. These findings highlight the necessity of monitoring duplicated resistance alleles in natural populations to formulate region-specific resistance management strategies.

**Supplementary Information:**

The online version contains supplementary material available at 10.1186/s40249-026-01421-0.

## Background

Malaria remains an important public health problem in tropical and sub-tropical countries. According to a report from the World Health Organization (WHO), there were approximately 263 million clinical cases of malaria and 597,000 deaths from malaria infection in 2023, with nearly 94% of them occurring in Africa [1]. It also ranked first among the top five important parasitic diseases in China in the past [2]. Despite the WHO's declaration in 2021 that China has successfully eliminated malaria [3], the presence of imported malaria cases presents a significant obstacle to maintaining sustainable control of the disease in the country.

*An. sinensis* is the most concerning vector for malaria transmission because of its wide distribution and species predominance in China [4]. The major breeding sites of *An. sinensis* in China are rice fields, where organophosphates (OPs) and carbamates (CBs) have been used intensively for agricultural pest control since the early 1950s [5]. Prolonged exposure has consequently generated variable levels of resistance to both insecticide classes across multiple regions of the country [6].

Acetylcholinesterase (AChE, EC 3.1.1.7) is a synaptic enzyme that hydrolyzes the neurotransmitter acetylcholine to terminate nerve impulses. OPs and CBs act as competitive inhibitors that irreversibly inhibit the AChE, thereby blocking nervous transmission and causing the death of the insect. In the mosquito genome, two paralogous genes—*ace-1* and *ace-2*—encode AChEs and only *ace-1* has been consistently implicated in resistance to OPs and CBs [7]. Resistant insects often have mutations in the *ace-1* gene that reduce the binding affinity of these insecticides, allowing the enzyme to function normally despite the presence of the chemical. To date, three non-synonymous point mutations (G119S, F290V and F331W) have been associated with insecticide resistance in mosquitoes [8]. However, multiple lines of evidence have revealed that only one, G119S mutation (*Torpedo californica* numbering), has been found in *Anopheles* [9–14]. This mutation causes a spatial shift of AChE1 structure, which reduces insecticide binding affinity and simultaneously lowers catalytic activity by > 60% relative to the susceptible enzyme [15, 16]. Consequently, 119S homozygotes exhibit substantial fitness costs in insecticide-free environments, whereas heterozygotes incur comparatively milder penalties [17, 18]. This resistance–fitness trade-off drives spatial and temporal fluctuations in 119G/119S allele frequencies and has further promoted *ace-1* polymorphism.

Currently, next-generation sequencing (NGS) technologies have revealed that copy-number variations (CNVs), such as deletions and duplications of genetic material, are widespread in natural populations. CNVs in *ace-1* gene have also been discovered in *An. gambiae s.l.* and *Culex pipiens* subspecies which suggests a role of CNVs in *ace-1* gene in resistance to OPs and CBs [18]. The G119S mutation is well documented in *An. sinensis* field populations across China [5, 13, 19], CNVs in the *ace-1* gene have not yet been reported in this species to date.

Guizhou Province, located in southwest China, was once one of the provinces with the highest malaria burden (88.97/10,000) [20]. However, there is a paucity of reported information on the insecticide resistance status of *An. sinensis*. In this study, the resistance status of *An. sinensis* field populations across Guizhou Province was first characterized by detecting the distribution and prevalence of the *ace-1* resistant allele and residual AChE activity. Subsequently, CNVs in the *ace-1* gene were identified through genome scanning and standard genomic DNA (gDNA) quantitative PCR (qPCR). Notably, heterogeneous duplication of the *ace-1* gene was identified in this species for the first time globally.

## Methods

### Mosquito samples

Based on the historical prevalence of malaria in Guizhou Province, we selected 12 sampling sites. Adult mosquitoes were collected at these sites from 2017 to 2024 using ultraviolet light traps (wavelength: 365 nm). The collection information and the codes for all 12 mosquito populations are provided in Table 1. In each site, three or four houses (with a distance > 50 m) were equipped with a light trap per house. The light trap was placed in the pig pen or cattle pen located near the rice field, 1.5 − 2.0 m above the ground. The mosquitoes were collected from 1 h before sunset to 1 h after sunrise, for three consecutive days. After morphological identification, confirmed adult *An. sinensis* mosquitoes were assigned to different preservation protocols based on the intended analysis. Specimens for molecular detection were stored in Eppendorf tubes containing 100% ethanol at − 4 °C, whereas those for enzymatic activity assays were preserved in cryotubes within a liquid nitrogen container.

**Table 1:** Collection information and population codes for field *Anopheles sinensis* in Guizhou Province, China

Collection sites	Longitude and latitude	Altitude	Collection date	Population code
DeJiang County, TongRen City	28.319 N, 108.142 E	560 m	2018.08–2020.07	DJ
XiShui County, ZunYi City	28.156 N, 106.348 E	402 m	2018.08–2020.07	XS
TongZi County, ZunYi City	28.115 N, 106.595 E	1018 m	2018.08–2020.07	TZ
PingBa County, AnShun City	26.554 N, 106.341 E	1253 m	2017.07–2018.07	PB
HuaXi District, GuiYang City	26.399 N, 106.591 E	1170 m	2018.07–2020.07	HX
ZhiJin County, BiJie City	26.694 N, 105.835 E	1270 m	2018.07–2019.07	ZJ
LiPing County, QianDongNan Prefecture	26.202 N, 108.951 E	594 m	2018.07–2020.07	LP
DuYun City, QianNan Prefecture	26.292 N, 107.439 E	842 m	2018.07–2019.07	DY
XingRen City, QianXinan Prefecture	25.411 N, 105.230 E	1341 m	2018.08–2020.07	XR
SanDu County, QianNan Prefecture	25.572 N, 107.933 E	485 m	2018.07–2020.07	SD
LuoDian County, QianNan Prefecture	25.226 N, 106.682 E	389 m	2017.08–2020.07	LD
CeHeng County, QianXinan Prefecture	24.994 N, 105.771 E	706 m	2017.08–2024.07	CH

### Extraction of genomic DNA and species identification

The genomic DNA of a single mosquito was extracted with TaKaRa MiniBEST Universal Genomic DNA Extraction Kit (Ver. 5.0, TaKaRa Bio Engineering (Dalian) Co., Ltd., Dalian, China) following the manufacturer’s instructions. All extractions were performed in a designated separate room. Genomic DNA was kept at − 20℃ until use. The extracted DNA was used immediately for the PCR assay or stored at − 20℃ for later use. Molecular identification of *An.sinensis* species was performed with primers for mitochondrial DNA cytochrome oxidase subunit I (MT-CO1) [21]. The MT-CO1 gene fragment was amplified in a 25 μl reaction system containing 12.5 μl Premix Taq™ (TAKARA Bio Inc., Shiga, Japan), 8.5 μl ddH2O, 2 μl DNA template and 1 μl each of 10 μmol/L (As-mtDNA-COIF and As-mtDNA-COIR) primers (see S1 Table). PCR reactions were performed in a SimpliAmp™ Thermal Cycler (Thermo Fisher Scientific Inc., Waltham, MA, USA) under the following conditions: 95 °C for 3 min, followed by 35 cycles of 95 °C for 30 s, 55 °C for 30 s, and 72 °C for 30 s, and terminated with a final extension at 72 °C for 10 min. PCR products (5 μl) were identified and bi-directionally sequenced by Sangon Biotech (Shanghai, China).

### Amplification and sequencing of *ace-1*

A 193 bp DNA fragment of the *ace-1* gene was amplified in a 25 μl reaction system containing 12.5 μl Premix Taq™ (TAKARA Bio Inc., Shiga, Japan), 8.5 μl ddH_2_O, 2 μl DNA template and 1 μl each of 10 μmol/L (As-ace1F and As-ace1R) primers (see S1 Table). The reactions were performed in a SimpliAmp™ Thermal Cycler (Thermo Fisher Scientific Inc., Waltham, MA, USA) under the following conditions: 94 ℃ for 3 min, followed by 35 cycles of 95 ℃ for 30 s, 55 ℃ for 30 s, and 72℃ for 45 s, and a final extension at 72 ℃ for 10 min. A non-template control (NTC) and a positive control were included in each run. All PCR setups were conducted in a Class II biosafety cabinet, and post-PCR analysis was performed in a designated separate room to prevent contamination. PCR products (5 μl) were identified and bi-directionally sequenced by Sangon Biotech (Shanghai, China).

### Biochemical assay to measure AChE1 residual activity

AChE1 residual activity was estimated in the presence of inhibitor (propoxur) with AChE activity assay kit (Beijing Solarbio Science & Technology Co., Ltd, Beijing, China) with some modifications. LP population was designated as the susceptible population based on its low frequency (12%) of *ace-1* mutant genotypes, which predicts high susceptibility to OPs and CBs. The procedure was described as follows.

For each replicate, a pool of four de-abdomened mosquitoes was homogenized on ice in 400 μl of tissue extraction buffer, with at least four replicates per population. The tissue homogenates were centrifuged at 8000 × *g*, 4 ℃ for 10 min and 15 μl of the supernatant was dispensed into each of two tubes labeled A and B. We added 10 μl ethanol (95%) to tube A and 10 μl of propoxur (a CB insecticide, at 10^–1^ mol/L diluted in ethanol) to tube B. Both tubes were incubated for 15 min at room temperature. Subsequently, 50 μl of substrate solution (acetylthiocholine iodide) was added to each well and incubated them at 37 ℃ water bath for 5 min. Then 50 μl of stop solution (trichloroacetic acid) was added and centrifuged at 12,000 × *g* for 5 min at room temperature. After that, 10 μl of mixture was dispensed into each of two wells of a 96-well microtitration plate and 170 μl of dilution buffer (citrate buffer) and 20 μl of staining solution (DTNB) were added. At last, AChE1 residual activity was estimated by measuring the change in optical density at 412 nm with Biotek epoch2 microplate reader (Bio-Tek Instruments, Inc., U.S.A.) following the cleavage of acetylthiocholine, as described by Ellman et al. [22]. OD value of the first well (ethanol) was set as OD_total_, whereas OD value of the second (propoxur) as OD_inhibitory_. For detected populations, four to six replicates were imperative. The AChE1 residual activity was calculated by the equation in the kit listed below. For comparisons of different populations, samples of each were distributed on the same plate and analyzed simultaneously to avoid experimental artifacts.

AchE residual enzyme activity (U/g) =  [ΔA ÷ (ε × d) × V_coloration_ × 10^9^] ÷ (W × V_sample_ ÷ V_total sample_ × V_supernatant_ ÷ V_enzyme_) ÷ T = 2255 × ΔA ÷ W.

ε: Molar Extinction Coefficient; ΔA = OD_total_—OD_inhibitory_; d: Optical path of cuvette; V_coloration_: total volume of coloration; 10^9^: unit conversion factor; V_sample_: volume of sample; W: total weight of sample; V_total:_ total volume of sample; V_supernatant:_ volume of supernatant; V_enzyme_: total volume of enzyme reaction; T: time of reaction.

### Whole-genome sequencing

Genomic DNA was extracted from 20 individual female mosquitoes of the LP (5 samples), CH (4 samples), HX (4 samples), TZ (2 samples) and DJ (5 samples) populations with the Qiagen DNeasy kit and was treated with RNase A to remove residual RNA. DNA concentration was assessed in the Qbit dsDNA BR Assay (Thermo Fisher Scientific,Waltham, Massachusetts, USA). Illumina whole-genome sequencing libraries were constructed with the Nextera DNA sample preparation kit (Illumina,Waltham, Massachusetts, USA), in accordance with the manufacturer’s instructions. FASTQ format libraries were generated from 150 bp-read pairs separated by a 300 bp insert. After BLAST comparison search, similar sequences were found only in *An. sinensis* China (GCA_000441895.2). Therefore, the *An. sinensis* China genomic assembly was selected for subsequent analysis [23]. The *An. sinensis* China genomic assembly did not obtain complete chromosome sequences, but instead contained 19,184 sequence fragments of different sizes. Unfortunately, the fragment size of the sequence KE524393, where *ace-1* is located, is only 41,288 bp, and the chromosomal location of the fragment is not mentioned, so it was impossible to use adjacent fragments to identify the presence of copy number variant repeats in this region. Therefore, the non-adjacent fragment KE525266, where the voltage-gated sodium channel gene (*vgsc*) is located, was considered to use for determining whether a copy number duplication exists in the *ace-1* gene. Bowtie 2 software (version v 2.3.5.1, https://sourceforge.net/projects/bowtie-bio/files/bowtie2/) was used to map the readings to the *An. sinensis* China dataset. The open-source software Samtools (https://github.com/samtools/samtools) and bedtools (Quinlan laboratory, University of Utah,USA) was used to calculate depth of coverage (DOC). Excel 2016 was used (Microsoft, USA) to calculate DOC ratios for different populations and R v4.1 (https://cloud.r-project.org/) was used to draw the image.

### Duplication detection and characterization

A three-step approach was used to detect and characterize the structure of the duplication either containing the *ace-1* locus or *vgsc* locus. Here *vgsc* was a reference locus as a single copy. LP population was a sensitive population, while the other 4 populations were resistant populations. First, the variations in read DOC for short-read mappings on the genome sequence KE524393 (*ace-1*) and KE525266 (*vgsc*) at different locations were analyzed. This made it possible to detect CNVs; Duplications induce a local increase in DOC, whereas deletions induce a local decrease in DOC compared to single copy locus. Then, the DOC of each base was calculated by determining the read depth of the segment falling within the 100 bp sliding window. This makes it possible to minimize the computer resources required without losing DOC information. Finally, DOC shifts were detected by calculating the ratio of DOC between resistant and sensitive populations.

### Gene copy-number quantification

The number of copies present for *ace-1* relative to *rps7* gene present as a single copy in the VectorBase PEST genome was estimated by standard genomic DNA quantitative PCR (standard gDNA qPCR, Biorad Cop, U.S.A) [18]. A 20 μl qPCR reaction was prepared in each well of a 96-well plate. The reaction consisted of 2 μl of genomic DNA and 18 μl of a master mix containing 10 μl of MCE qPCR SYBR MIX (Takara, Japan), 1.6 μl of gene-specific primers (0.5 μmol/L final concentration each), and 6.4 μl of ddH_2_O. The specific primers for both the *ace-1* gene (As-ace1qF, As-ace1qR) and the *rps7* gene (As-rps7qF, As-rps7qR) were listed in S1 Table. It was performed as follows: activation at 95 ℃ for 15 min, followed by 40 cycles of 95 ℃ for 15 s, 60 ℃ for 45 s, and 72 ℃ for 19 s. Melting curves were generated by a post-amplification melting step between 70 ℃ and 95 ℃, for T_m_ analysis. All quantification was replicated four times for each DNA template. Standard curves were constructed with tenfold dilutions of a PCR product previously amplified with specific primers for both genes from LP population DNA. The *ace-1* gene concentration ratios over *rps7* were determined by the double-standard curves method. Finally, the mean value of the number of copies of the *ace-1* relative to the *rps7* was calculated for each region.

### Data analysis

The sequencing data was manually checked and cleaned with Chromas 2.6.5 (Technelysium Pty Ltd, Australia). All confirmed DNA sequences were aligned with the sequence at locus 119 in *ace-1* in the MEGA 11.0 software (https://www.megasoftware.net/, Sudhir Kumar, USA.). The nucleotide and amino-acid sequences of *ace-1* (Genbank ID: MG953803.1) of *An. sinensis* were used as the reference for alignment. Nucleotide variations were documented in Excel 2016 (Microsoft, USA.). Statistical comparisons of *ace-1* copy numbers between the LP population (designated as the susceptible control) and each field population were performed using two independent-sample *t*-test. A chi-squared test was carried out to compare the overall difference in the allele frequency and genotype frequency among *An. sinensis* populations by IBM SPSS 22.0 (Statistical software, International Business Machines Corporation, USA). Hardy–Weinberg equilibrium (HWE) was estimated using Arlequin 3.5 (Genetic data analysis software, L. Excoffi, CMPG, University of Berne, Switzerland).

## Results

### The distribution and frequency of alleles and genotypes in the *ace-1 *gene among *An. sinensis* populations

**Fig. 1:**
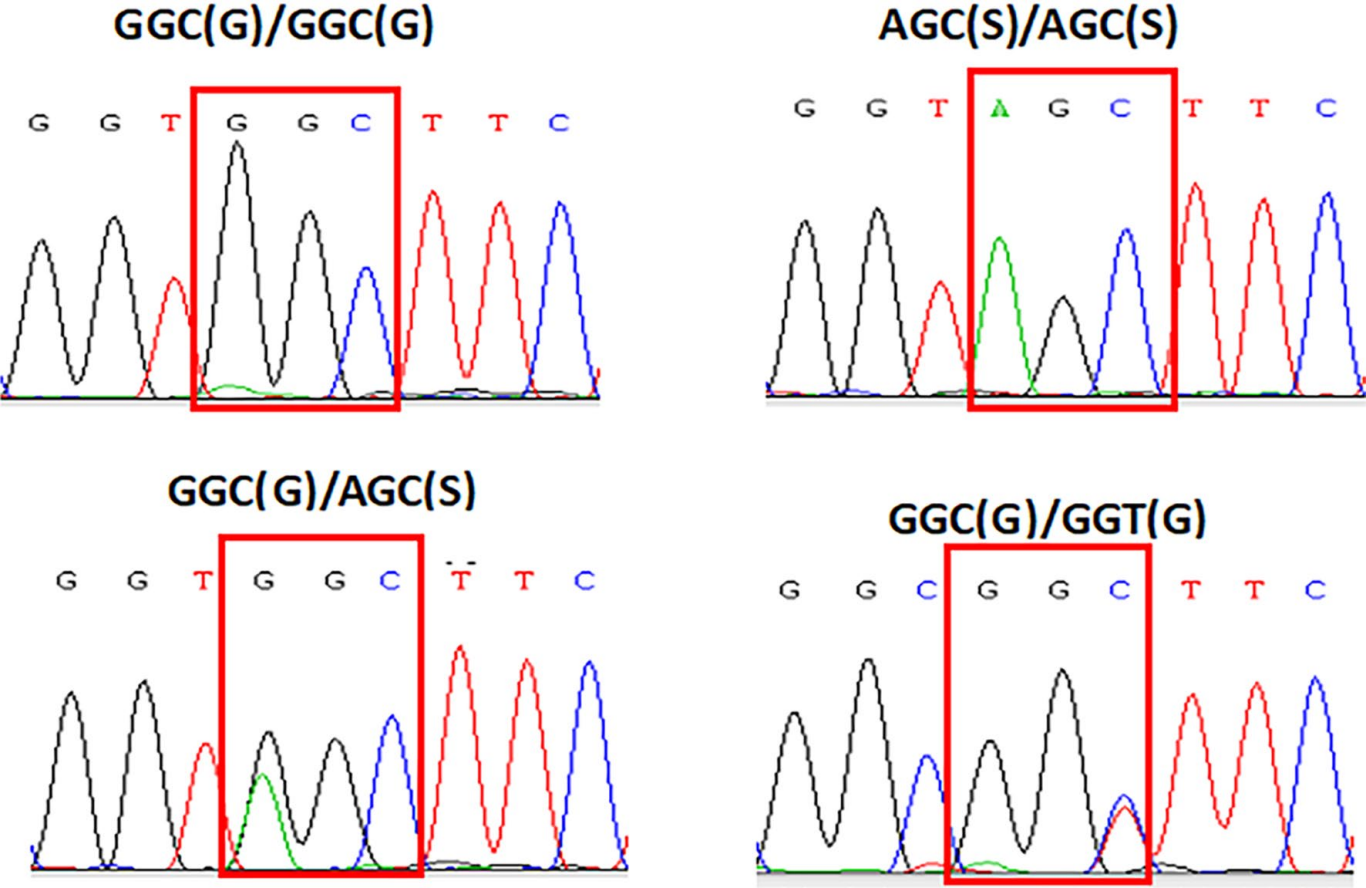
Nucleotide sequence chromatograms showing the four detected genotypes of the ace-1 gene in *Anopheles sinensis* populations from Guizhou Province, China. Codon 119 (the site of the critical mutation) is highlighted with a box in each chromatogram

Three alleles (GGC/G, GGT/G and AGC/S) were identified at the locus 119 of *ace-1* in 551 samples of *An. sinensis* from the 12 populations (Fig. 1)*.* The GGT/G allele was found for the first time and in only one sample of the ZJ population. The average frequency of the resistance allele (AGC/S) was 50.36% in the 12 populations. Specifically, the frequency of the resistance allele AGC/S ranged 6% (LP population) –77% (CH population) among the 12 populations (Table 2). Chi-square test indicated the alleles varied significantly among the 12 populations (*χ*^2^ = 128.02, *P* < 0.001).

**Table 2:** Distribution and frequency of genotypes and alleles at 119 locus of the *ace-1* gene in *Anopheles sinensis* from Guizhou Province, China

Populations	*n*	Genotypes frequency (%)			HWE test (*P* value)	Alleles frequency (%)**		
		GG	SS	GS	Probability test	GGC/G	GGT/G	AGC/S
LP	50	88	0	12	1.000	94	0	6
CH*	50	0	34	66	< 0.001	33	0	77
LD	25	0	40	60	0.060	30	0	70
SD*	49	0	22	78	< 0.001	38.78	0	61.22
XR	51	8	43	49	0.528	32.35	0	67.65
PB	50	24	14	62	0.149	55	0	45
ZJ	50	16	26	58	0.388	43	2	55
HX	50	22	18	60	0.257	52	0	48
DY*	50	34	4	62	< 0.05	65	0	35
XS*	26	4	12	85	< 0.001	46.15	0	53.85
TZ*	50	4	4	92	< 0.001	50	0	50
DJ*	50	4	18	78	< 0.001	43	0	57
Average	-	17	19.58	63.5	-	49.47	0.17	50.36

^*^Populations showed a significant deviation from the Hardy–Weinberg equilibrium at *P* < 0.05

^**^ Chi-square test:* χ*^2^ = 128.02, *df* = 11, *P* < 0.001

The frequency of mutant genotypes (119SS, 119GS) was over 66% in all 12 field populations, with the exception of the LP population, where it was 12%, suggesting this population remains largely susceptible (Table 2). Therefore, we classified the LP population as an OPs and CBs sensitive population, while the remaining populations were designated as resistant populations throughout the remainder of this paper. Among them, a 100% frequency of mutation genotypes was observed in the populations of CH, LD and SD, which were from the once highly epidemic areas of malaria, and the frequency of that in 4 populations (DJ, XS, TZ and XR) was more than 90%. The heterozygote 119GS was the predominant genotype and there was a significant deviation from the HWE at this locus in half of 12 populations (Table 2), which indicated a heterozygote excess in the field populations.

### AChE1 residual activity

**Fig. 2:**
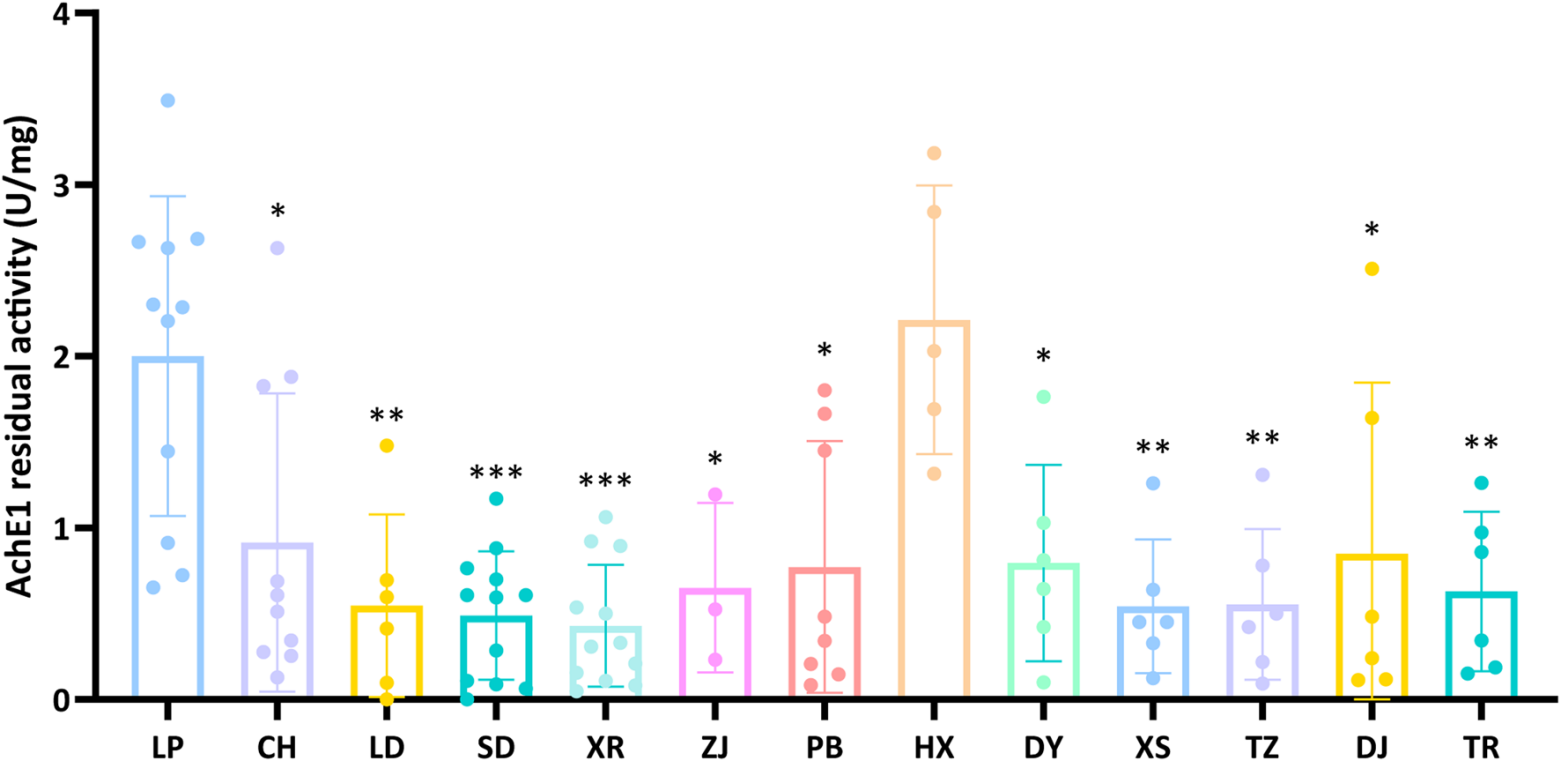
Comparison of the residual acetylcholinesterase 1 (AChE1) activity in 12 *Anopheles sinensis* populations from Guizhou Province, China. Statistical significance was determined using the two independent-sample t-test,with significance levels indicated as *P* < 0.05 (*), *P* < 0.01 (**), *P* < 0.001 (***)

As shown in Fig. 2, when propoxur was used as an inhibitor, the residual AChE1 activity in most populations showed a significant reduction compared to the LP population (*t* = 2.370 to 5.433, *P* < 0.05), with the exception of the HX population (*t* = 0.4406, *P* = 0.6663). These findings provided further evidence that OPs and CBs resistance was widespread among *An. sinensis* populations in Guizhou Province.

### Heterogeneous duplication of *ace-1* existed in the genomic structure of *An. sinensis*

**Fig. 3:**
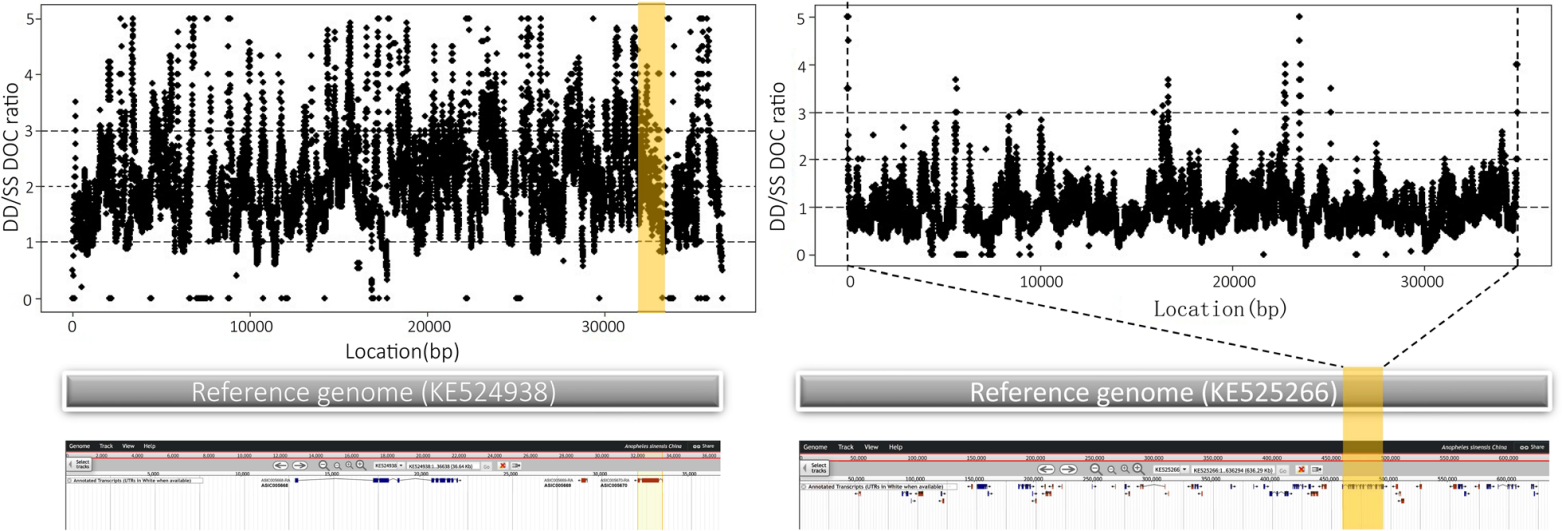
Copy number variation (CNV) analysis of the *ace-1* and *vgsc* genes based on sequencing depth in the CH (CeHeng) and LP (LiPing) populations of *Anopheles sinensis*. **A** The depth of coverage (DOC) ratio of the *ace-1* gene in the CH and LP population. **B** The depth of coverage (DOC) ration of the vgsc gene in the CH and LP population. The position of the yellow box represents the location of the *ace-1* and *vgsc* gene in the corresponding genome. Gbrowse view of the duplicated region in VectorBase (https://www.vectorbase.org)

Given the heterozygote excess for the *ace-1* gene observed in 6 populations, we further characterized the genomic structure of the *ace-1* gene. A heterogeneous duplication (designated as the D allele, carrying one copy of the S allele and one copy of the R allele), was firstly identified in the field population genome of *An. sinensis* by comparing the genomes of LP (sensitive population) and CH (resistant population) populations. The average DOC ratio of the genomic region containing the *ace-1* gene between the CH and LP populations was approximately 2.25, whereas the average DOC ratio of the genomic region containing the *vgsc* gene between these two populations was about 0.963 (Fig. 3). Meanwhile, we also determined the average DOC ratios of the *ace-1*-containing genomic region between the DJ/HX/TZ populations and the LP population, which mainly fluctuated around 2; in contrast, the average DOC ratios of the *vgsc*-containing genomic region under the same comparison mainly fluctuated around 1.

### The copy number of the *ace-1* gene in field populations.

**Fig. 4:**
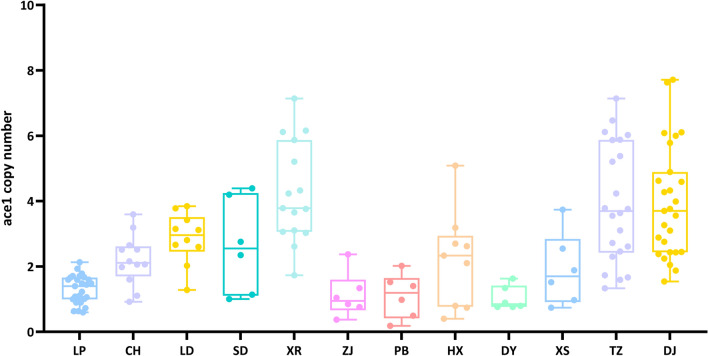
Mean *ace-1* copy number in 12 populations of *Anopheles sinensis* in Guizhou Province by standard gDNA qPCR

Finally, there was a substantial variation in the mean copy numbers of the *ace-1* gene among the different field populations (Fig. 4, Stable 2). The LP population exhibited a mean copy number of approximately 1, while the CH, LD, SD, TZ, and DJ populations showed mean copy numbers around 2. The considerable variation might be related to the field individual mosquito. This observed variation may reflect inherent differences among individual mosquitoes in field populations. It should be noted, however, that our current data do not provide information about the distribution of these copies across individual chromosomes.

## Discussion

The mosquito *An. sinensis* is the most important malaria vector in China and other Southeast Asian countries. Based on historical malaria epidemic distribution patterns, we selected 12 field populations of *An. sinensis* across Guizhou Province in this study. Genotyping analysis of the *ace-1* gene revealed a moderate frequency (50.36%) of the 119S resistance allele in these populations. Previous studies on *An. sinensis* in other regions of China, including Shanghai (85.98%) [13], Sichuan (56%) [19], Guangxi Zhuang Autonomous Region (80%) [24], the Chinese side of the China-Vietnam border (73%) [25], Hainan Island (45–75%) [26], Yunnan (38.5%), and Anhui (58.9%) [27], have documented moderate-to-high frequencies of the 119S allele. Collectively, these findings indicate that the G119S mutation is widespread in *An. sinensis* field populations across China. The G119S substitution (glycine to serine) in the *ace-1* gene is well known to confer very strong resistance to OPs and CBs at both larval and adult stages in *Anopheles* spp. and *Culex* spp. [18]. To assess the functional impact of this mutation in *An. sinensis* from Guizhou, we measured the residual activity of AChE1 in the presence of propoxur across all populations. The LP population was classified as a sensitive population in this study, as it exhibited a low frequency of *ace-1* mutant genotypes (12%). In contrast, most populations with high frequencies of the resistance allele showed clear propoxur resistance, indicating consistency between genotyping and biochemical results. The widespread use of OPs and CBs in Chinese agriculture since the 1950s is likely responsible for the high prevalence of mutant genotypes observed in these mosquito populations [26]. Notably, mutant genotypes (119GS, 119SS) were observed at frequencies > 90% not only in three regions of Guizhou—CH, LD, and SD—previously considered the highest malaria-endemic areas in the province but also in three regions of northern Guizhou (XS, TZ and DJ) bordering Sichuan. This further indicates a high risk of resistance to OPs and CBs in these regions.

Previous studies have consistently identified *ace-1* heterozygotes as the predominant genotype in field populations across multiple regions of China [13, 19, 24–28]. However, HWE tests for this locus have been limited to populations from Sichuan Province [19] and Guangxi Zhuang Autonomous Region [24, 25]. Notably, significant deviations from HWE were observed in some *An. sinensis* populations from Sichuan. In the present study, *ace-1* heterozygote was also the predominant genotype and a significant deviation from HWE was observed in six of the 12 *An. sinensis* field populations, including the CH and SD populations. These deviations, likely driven by excess heterozygosity, suggest that a heterozygote advantage may exist in natural *An. sinensis* populations under insecticide pressure in Guizhou and Sichuan. Such an advantage implies an associated fitness cost of the resistant allele, a phenomenon previously documented in *An. gambiae s.l.* and *Culex pipiens * [18]. A heterogeneous duplication of the *ace-1* gene—D allele, where a susceptible S allele and a resistant R allele are carried on the same chromosome—creates permanent "heterozygotes." This genotype confers resistance to OPs and CBs, similar to conventional heterozygotes, but with reduced fitness costs [29, 30], and has been recognized as a key contributor to insecticide resistance in *An. gambiae s.l.* and *Cx. pipiens* [31, 32]. To investigate this in *An. sinensis* populations, whole-genome comparisons were performed between 5 *An. sinensis* populations (CH, DJ, HX, TZ, and LP populations) and the reference genome. Strikingly, the CH, DJ, HX, and TZ populations exhibited approximately twice the *ace-1* copy number of the LP population, with no duplication detected in the *vgsc* gene. These findings were validated by standard gDNA qPCR, representing the first report of the *ace-1* gene heterogeneous duplication in *An. sinensis* and revealing a novel resistance mechanism in this vector species. Additionally, both heterogeneous duplications and homogeneous amplifications strongly associated with resistance to OPs and CBs have been widely documented in *An. gambiae s.l.* populations in West Africa [33]. This indicates that CNVs in the Anopheles genome are more complex and potentially of great medical significance.

Meanwhile, two key observations highlight the greater-than-anticipated complexity and heterogeneity of fitness costs associated with the *ace-1* resistance allele in *An. sinensis*: the fixation of the resistant homozygote (119SS) in the *An. sinensis* population from Wenzhou City [34], and the absence of HWE deviations among predominantly heterozygous individuals in the populations from Guangxi [24, 25]. While these findings could potentially be attributed to sampling limitations, they nevertheless emphasize the need to further investigation. A more comprehensive investigation on *ace-1* gene polymorphism in *An. sinensis* will require integrating single nucleotide polymorphism analysis with genome-wide scans.

Several limitations should be acknowledged when interpreting the findings of this study. First, whole-genome sequencing was performed on a limited sample size of 20 female mosquitoes from five populations, which failed to encompass the genetic diversity of all 12 sampled populations across Guizhou Province. This may result in biases when inferring the distribution characteristics of *ace-1* gene copy number variations. It is clear that the utilization of the *An. sinensis* reference genome (GCA_000441895.2) that has not yet been assembled at the chromosome level has resulted in certain limitations. The fragment containing the *ace-1* gene (KE524393) has a length of 41,288 bp, and its precise chromosomal location remains to be elucidated. This limitation prevented the study from verifying the boundaries, continuity, and flanking sequence characteristics of the *ace-1* gene duplication region through alignment with adjacent genomic fragments, potentially affecting the accurate resolution of the complete structure of heterogeneous duplication events. Lastly, the study did not investigate other potential resistance mechanisms, such as metabolic detoxification or behavioral avoidance, which may also play significant roles in field resistance.

## Conclusions

Resistance to OPs and CBs is widespread in *An. sinensis* populations across Guizhou Province. For the first time, a heterogenous duplication has been identified in *An. sinensis* mosquito. This duplication, along with point mutation of G119S, confers the resistance to OPs and CBs in *An. sinensis* in Guizhou Province. Continued monitoring of duplicated resistant alleles in natural populations is essential for developing effective region-customized resistance management strategies for sustainable malaria control in China.

## Supplementary Information

Additional file 1. Stable 1 The primers in this study.

Additional file 2. Stable 2 Statistical analysis of the ace-1 gene copy numbers in 12 field populations of Anopheles sinensis from Guizhou Province. SFig. 1 The duplication event of ace-1 and vgsc based on the depth of coverage (DOC) ratio (DJ/HX/TZ population/LP population). The average DOC ratio of ace-1 of the points mainly fluctuated around 2.35, 1.96 and 1.72. 2 And the average DOC radio of vgsc mainly fluctuated around and 1.16, 1.45 and 0.78.

## Data Availability

The raw sequencing data generated in this study have been deposited in the NCBI Sequence Read Archive (SRA) under BioProject accession ON257305–ON257855.

## Abbreviations

Ops: Organophosphates
CBs: Carbamates
AChE: Acetylcholinesterase
CNVs: Copy-number variations
mtDNA-COI: Mitochondrial cytochrome oxidase subunit I
gDNA qPCR: Genomic DNA quantitative PCR
