# Supplementary material for: Heterogeneous duplication and point mutation of ace-1 confer organophosphates and carbamates resistance to Anopheles sinensis field populations in Guizhou Province, China
Source: Infect Dis Poverty. 2026 Mar 9;15:32. doi: 10.1186/s40249-026-01421-0 (PMC12969906; doi:10.1186/s40249-026-01421-0)
Supplement: Supplementary file 1 — Additional file 1. Stable 1 The primers in this study. [file 40249_2026_1421_MOESM1_ESM.docx]

| Tab. 1 primers in this study | | | |
| --- | --- | --- | --- |
| Primer Name | primer sequence （5'-3'） | bp | Gene Accession Number |
| mtDNA-COIF | GGTCAACAAATCATAAAGATATTGG | 709 | - |
| mtDNA-COIR | TAAACTTCAGGGTGACCAAAAAATCA |  |  |
| ACE1-F | GCGCGACCATGTGGAACC | 193 | KU900233 |
| ACE1-R | ACCACGATCACGTTCTCCTC |  |  |
| As-ace1qF | TCAACAACGAGTGGGGTACG | 187 | KU900233 |
| As-ace1qR | AGCAGCTCGGTCAGGTAGTA |  |  |
| As-rps7qF | TGGAGAGCGTCGTATTCTGC | 124 | LOC131289104 |
| As-rps7qR | CGGGAACACCAAATCCTCCA |  |  |
